# Pseudogene HSPA7 is a poor prognostic biomarker in Kidney Renal Clear Cell Carcinoma (KIRC) and correlated with immune infiltrates

**DOI:** 10.1186/s12935-021-02141-1

**Published:** 2021-08-19

**Authors:** Chunjin Ding, Rundong He, Jinghan Zhang, Zhan Dong, Jun Wu

**Affiliations:** 1 Department of Orthopaedics, Affiliated Haian Hospital of Nantong University, Nantong, Jiangsu China; 2 The Research Center for Bone and Stem Cells, Department of Anatomy, Histology and Embryology, Nanjing Medical University, Nanjing, Jiangsu China; 3 Neonatal Medical Center, Children’s Hospital of Nanjing Medical University, Nanjing, Jiangsu China; 4 Department of Orthopaedics, Children’s Hospital of Nanjing Medical University, Nanjing, Jiangsu China

**Keywords:** Kidney Renal Clear Cell Carcinoma (KIRC), HSPA7, Immune, Prognostic biomarker, Tumor

## Abstract

**Background:**

Pseudogenes played important roles in tumorigenesis, while there are nearly no reports about the expression and roles of HSPA7 in the cancer.

**Methods:**

Firstly, we used Logistic regression, the KS test, the GEPIA database, UALCAN database and qRT-PCR to analyze the expression level of HSPA7 in KIRC, then we used the Cox regression and the Kaplan–Meier curve to analyze the overall survival (OS) of KIRC patients with different Clinico-pathological parameters. Thirdly, we used the multivariate Cox analysis of influencing factors to compare the correlation between the HSPA7 expression level and the clinical parameters. Finally, we used multi-GSEA analysis and the Tumor Immunoassay Resource (TIMER) database to explore the functional role of HSPA7 in KIRC

**Results:**

The HSPA7 is highly expressed in KIRC tumor tissues, and its expression is related to clinico-pathological features and survival in KIRC patients. GSEA analysis displayed the high expression of HSPA7 in KIRC were related to several tumor-related and immune-related pathways. With the TIMER database analysis we showed that HSPA7 levels were correlated with the CD4^+^ T cells, neutrophils and Dendritic Cell.

**Conclusions:**

Our study showed that HSPA7 is very important in the tumor progression and may act as a poor prognostic biomarker for KIRC tumor by modulating immune infiltrating cells.

**Supplementary Information:**

The online version contains supplementary material available at 10.1186/s12935-021-02141-1.

## Introduction

The morbidity of renal cell carcinoma (RCC) is about 4.2% of all newly-appeared cancer cases, which make RCC become one of the most frequent malignances worldwide. According to a recent survey, there were about 73,820 new cases of RCC and 14,770 deaths occurred in United States in 2019 [1]. Kidney Renal Clear Cell Carcinoma (KIRC) is the most common kidney cancer subtype [2]. At present, the primary treatment of KIRC is surgery, while 30% of the patients who underwent surgery still experience metastasis [3], and the currently used drugs are not effective and have relatively great side effects. Early identification and diagnosis of KIRC patients can help with more precise clinical treatment. Therefore, it is urgent to discover new and reliable markers to predict the prognosis of patients.

Pseudogenes are non-coding genes lacking of protein-coding ability, and were once labeled as junk genes. However, there is growing evidence indicated that pseudogenes can influence the regulatory mechanisms of many human cancers and pseudogene expression is treated as a novel marker and used in a variety of cancer types to stratify patients subtypes [4, 5] and is therefore taken into account in cancer survival prognostic factors. For example, the pseudogene PRELID1P6 can promote glioma progression through the hnHNPH1-Akt/mTOR pathway [6]. OCT4 abnormally activated pseudogene 5 (OCT4-pg5) can enhance cell proliferation by competing with miR-145 in endometrial carcinoma via upregulating OCT4 expression [7]. High expression of the pseudogene ANXA2P2 has been found to be related to a worse prognosis pseudogene in hepatocellular carcinoma [8]. LDHAP5 was associated with the poor prognosis of ovarian serous cystadenocarcinoma [9]. The Pseudogene HSPA7 (HSP70B) belongs to the HSP70 family (HSPA), discovered in 1985 and encoded near the highly homologous HSPA6 (HSP70B′) on chromosome 1, although mRNA can be expressed after thermal stimulation, it cannot transcribe a functional protein [10]. Numerous investigations have shown that HSPA6 plays an important role in multiple human cancers, including esophageal cancer [11, 12], glioma [13], lung cancer [14], hepatocellular carcinoma [15] and leukemia [16]. However, little has been reported about the expression and role of HSPA7 in cancer. In this study, we reported that high expression of HSPA7 can indicate the poor prognosis of KIRC.

Our study examined the expression and prognostic value of HSPA7 in KIRC patients in the Cancer Genome Atlas (TCGA) and validated them in multiple independent cohorts. Moreover, GSEA [17] and Tumor Immunoassay Resource (TIMER) database [18] were used to assay the potential mechanisms of HSPA7 in KIRC. Our results implied that the functional role of HSPA7 in KIRC may through regulating immune cell infiltration.

## Methods

### Data mining and data collection

The KIRC data of TCGA consists of 72 normal tissues and 539 tumor samples, was acquired from the TCGA data portal (https://tcga-data.nci.nih.gov/tcga/). Clinical data pertaining to patients’ age, gender, survival, grade, stage, and recurred/progressed outcome were also acquired from the data portal. The dataset including mRNA expression counts and survival data with clinical information. The samples with missing expression data were excluded from the study. The dataset contains survival data with clinical information and mRNA expression counts. The samples with missing expression data were excluded from our study.

### Data analysis

The R-3.6.2 project was used to analysis the acquired data. Firstly, we used the Logistic regression and the KS test to analyze the relation between the HSPA7 gene expression and Clinico-pathological features. Then we used the Cox regression and the Kaplan–Meier curve to analyze the overall survival of KIRC patients with different Clinico-pathological parameters from TCGA data. Finally, we used the multivariate Cox analysis of influencing factors to compare the correlation between the HSPA7 expression level and the clinical parameters, such as age, gender grade, stage, T classification, N classification, and M classification, related to survival. The Cutoff Finder.2 was used to determine the cut-off value of HSPA7 expression.

### Gene set enrichment analysis (GSEA)

Gene Set Enrichment Analysis (GSEA) is a computational method that determines whether an a priori defined set of genes shows statistically significant between two biological expression states [17]. In our study, an ordered list of genes based on the pathways related to the HSPA7 expression level were generated by the GSEA, and then the significant differences between the high and low-level expression groups of HSPA7 were annotated. The multi-GSEA results and signaling pathway enrichment analysis of phenotypes and were ranked by normalized enrichment score (NES) and the nominal p-value.

### Analysis of TIMER database

The TIMER (https://cistrome.shinyapps.io/timer/) database is designed for analysing immune cell infiltrates in multiple cancers. This database can estimate tumour immune infiltration by macrophages, dendritic cells, CD4/CD8^+^ T cells, neutrophils, and B cells [19]. We used the TIMER database to assess the HSPA7 different expression levels in particular tumours, and then we explored the correlation between HSPA7 expression level and the degree of infiltration in particular immune cell subsets. We further explored the differences in patient survival as a function of gene expression or immune cell infiltration by Kaplan–Meier curve analyses.

### Analysis of GEPIA and UALCAN database

The GEPIA (http://gepia.cancer-pku.cn/index.html) database and UALCAN (http://ualcan.path.uab.edu) database can explore the association of mRNA expression level with overall survival (OS). We used these two database to explore the correlation between the HSPA7 expression and patient overall survival in KIRC.

### RNA extraction and qRT-PCR analysis

A total of 20 primary KIRC cancer tissues was collected from patients who had undergone surgery at the First Affiliated Hospital of Nanjing Medical University and the Second Affiliated Hospital of Nanjing Medical University. The study was approved by the Ethics Committee of Nanjing Medical University (Nanjing, Jiangsu, PR China), and it was performed in compliance with the Declaration of Helsinki Principles. The clinical information of the 20 KIRC patients was shown in Additional file 1: Table S1). Written informed consent was obtained for all patient samples. RNA extraction and qRT-PCR of the KIRC cancer tissues were performed as the product manual described (Cat# R312-01, Cat# Q131-02, Vazyme, China). The primers used in this study are purchased from Generay (Shanghai, China) and listed as follows.

- HSPA7-R: CATCCCCAAGGTGCAGAAGT;
- HSPA7-F: ACCATCCTCTCCACCTCCTC;
- GAPDH-R: GGGAGCCAAAAGGGTCAT;
- GAPDH-F: GAGTCCTTCCACGATACCAA.

## Results

### Characteristics of the of the patients

537 patients’ clinical data were acquired from TCGA, including the age, gender, Histological grade, TNM classification of KIRC (Table 1).

**Table 1:** Clinical characteristics of TCGA KIRC patients (*n* = 537)

Clinical characteristics	Total (537)	%
Age		
< 60	247	46.00
≥ 60	290	54.00
Gender		
Female	190	35.38
Male	347	64.62
Histologic grade		
G1–2	244	45.44
G3–4	285	53.07
Gx	5	0.93
NA	3	0.56
Stage		
I–II	326	60.71
III–IV	208	38.73
NA	3	0.56
T classification		
T1–2	344	64.06
T3–4	193	35.94
N classification		
N0	240	44.69
N1	17	3.17
Nx	280	52.14
M classification		
M0	426	79.33
M1	79	14.71
Mx	30	5.59
NA	2	0.37
Vital status		
Deceased	170	31.66
Living	367	68.34

### High HSPA7 mRNA expression in KIRC

**Fig. 1:**
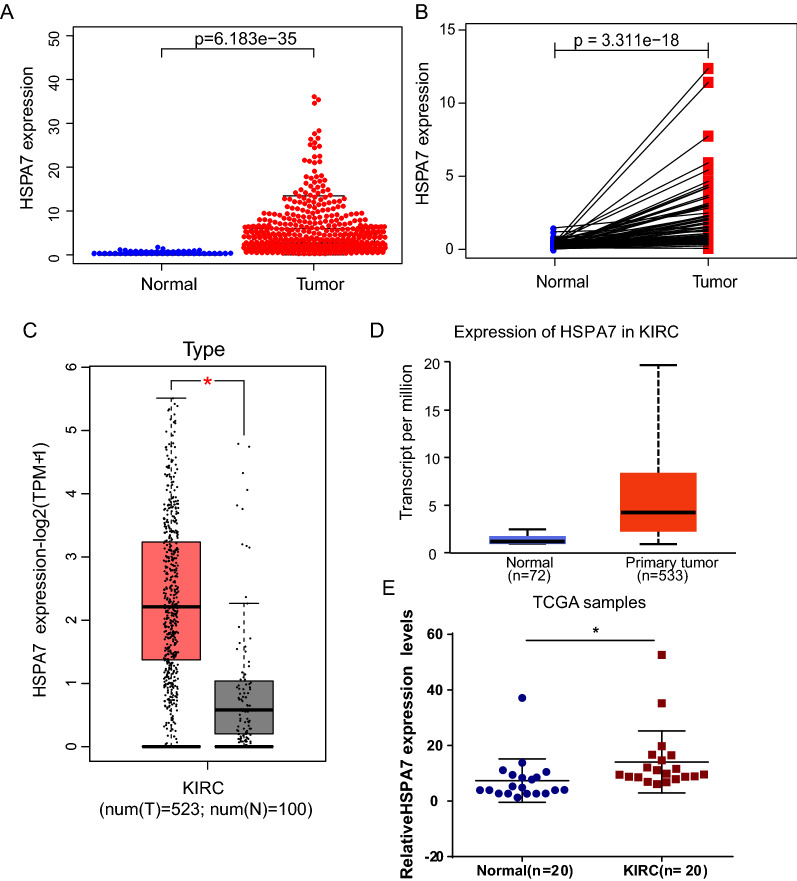
HSPA7 is overexpressed in KIRC. **A** HSPA7 mRNA expression in KIRC based on TCGA Data. **B** Paired difference analysis of HSPA7 mRNA expression in KIRC based on TCGA Data. **C** HSPA7 protein expression comparison between normal and tumor tissues obtained from the GEPIA web tool. **D** HSPA7 protein expression comparison between normal and tumor tissues obtained from the UALCAN web tool. **E** HSPA7 expression was inspected by qRT-PCR and normalized to GAPDH expression in human KIRC tissues compared with corresponding non-tumor tissues (n = 20) (*p < 0.05, log-rank test)

First, we assessed the differences in HSPA7 expression between KIRC tumor tissues and adjacent tissues via differential expression scatter plots and paired difference analyses. We find that the expression level of HSPA7 was significantly higher in KIRC tumor tissues (p = 6.183e−35) and in paired cancer tissues (p = 3.311e−18) compared with adjacent tissues (Fig. 1A, B). Then, the expression level of HSPA7 in KIRC tumor tissues and adjacent tissues were verified by GEPIA [20] (Fig. 1C) database, UALCAN database (Fig. 1D) [21] and qRT-PCR analysis (Fig. 1E). The clinical data of 20 patients’ used in qRT-PCR were shown in Additional file 1: Table S1.

### Correlation between HSPA7 expression level and clinico-pathological features in KIRC tumors

As the Table 2 shown the expression of HSPA7 was highly statistically significantly correlated with clinical stage (p = 0.044) and distant metastasis (positive vs. negative, p = 0.049).

**Table 2:** Correlation between the clinicopathologic characteristics and HSPA7 mRNA expressiona (logistic regression)

Clinical characteristics	Total (N)	Odds ratio in HSPA7 expression	p-value
Age (≥ 60 vs. < 60)	537	0.985 (0.700–1.386)	0.931
Grade (G1–2 vs.G3–4)	529	1.185 (0.840–1.673)	0.334
Stage (I–IV)	349	1.678 (1.018–2.794)	**0.044**
Local invasion (T1–2 vs. 3–4)	445	1.017 (1.002–1.032)	0.205
Distant metastasis (positive vs. negative)	505	1.636 (1.005–2.697)	**0.049**
Lymph nodes (positive vs. negative)	257	1.008 (0.360–2.825)	0.987

Bold values indicate statistically significant, p < 0.05

### Correlation between KIRC patients survival and HSPA7 expression

**Fig. 2:**
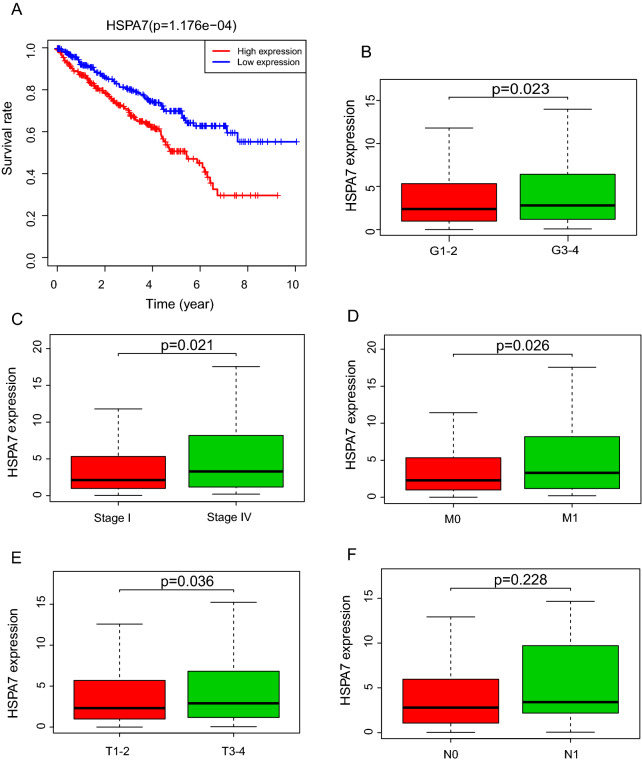
Correlation of HSPA7 Expression with Survival and clinicopathologic characteristics. **A** Survival analysis of HSPA7 expression in terms of overall survival. Kaplan–Meier curves produced survival analysis. **B** Subgroup analysis of Histological grade (G1–2 vs. G3–4). **C** Subgroup analysis of clinical stage (stage I and stage IV). **D** Subgroup analysis of M classification (M0 and M1). **E** Subgroup analysis of T classification (T1–2 and T3–T4). **F** Subgroup analysis of N classification (N0/N1)

**Fig. 3:**
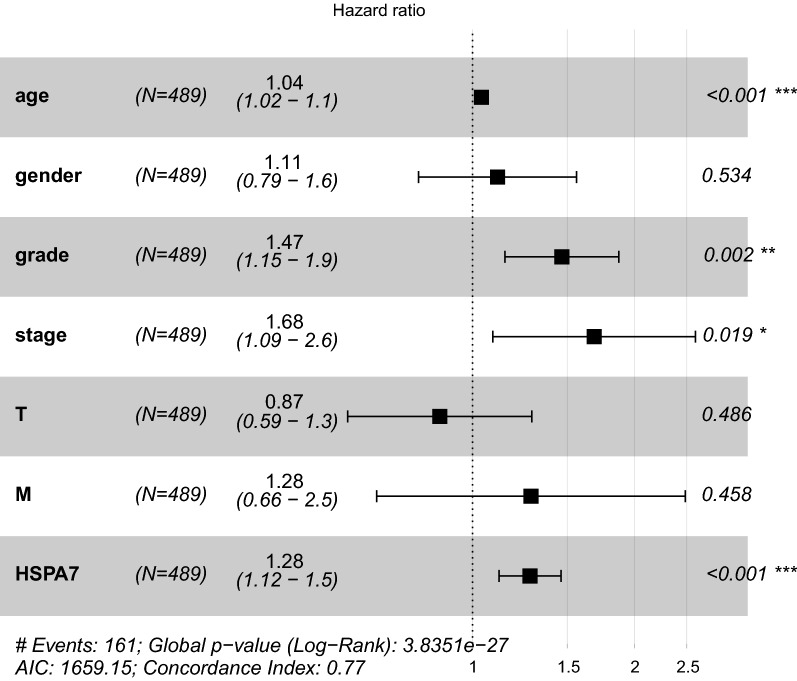
Forest map analysis of expression and clinicopathologic characteristics

To evaluate the effect of HSPA7 expression on KIRC patients survival, the log-rank test and Kaplan–Meier survival analysis were used to estimate the correlation between HSPA7 expression and KIRC patients prognosis. The patients with high HSPA7 expression level displayed relatively poor survival (p = 1.176e−04; Fig. 2A). The clinical subgroup analysis implied that the patients in Histological grade (G1–2 vs. G3–4), clinical stage (Stage I vs. Stage IV), M classification and T classification (the T1–2 vs. T3–4) with HSPA7 expression also had significantly poor overall survival (OS) (Fig. 2B–E), whereas not in the N classification (Fig. 2F). We performed the univariate analysis with the variables and listed in the Table 3. We also performed Multivariate analysis with the Cox proportional hazards model and the results implied that the expression of HSPA7 (HR = 1.304605, p = 0.005187) is a potential prognostic factor for KIRC patients (Table 4). Then we performed the forest plot analysis (Fig. 3), the outcome of KIRC patients are statistically significant correlation with age (p < 0.001), histological grade (p = 0.002), clinical stage (p = 0.019) and the expression of HSPA7 (p < 0.001). In conclusion, HSPA7 is a reliable and effective independent prognostic biomarker of KIRC patients.

**Table 3:** Univariate cox regression of overall survival and clinicopathologic characteristics in TCGA KIRC patients

Clinical characteristics (univariate cox regression)	Hazard ratio	HR (95% CI)	p-value
Age	1.022636	1.004910–1.040674	**0.012106**
Gender	1.013224	0.666050–1.541361	0.951059
Grade	2.242118	1.682289–2.988246	**3.61E−08**
Stage	1.862243	1.540797–2.250751	**1.26E−10**
T	1.943172	1.537502–2.455879	**2.69E−08**
M	4.073388	2.633544–6.300444	**2.76E-10**
HSPA7	1.046542	1.017951–1.075936	**0.001287**

Bold values indicate statistically significant, p < 0.05

**Table 4:** Multivariate analyses of overall survival and clinicopathologic characteristics in TCGA KIRC patients

Clinical characteristics (univariate cox regression)	Hazard ratio	HR (95% CI)	p-value
Age	1.031079	1.011672–1.050858	**0.001594**
Gender	1.329053	0.843200–2.094854	0.220453
Grade	1.439203	1.025090–2.020609	**0.035457**
Stage	1.485765	0.894490–2.467884	0.126193
T	0.962168	0.600494–1.541677	0.872618
M	1.700401	0.758832–3.810280	0.197200
HSPA7	1.304605	1.082694–1.572000	**0.005187**

Bold values indicate statistically significant, p < 0.05

### HSPA7-related signaling pathway was identified by GSEA

**Fig. 4:**
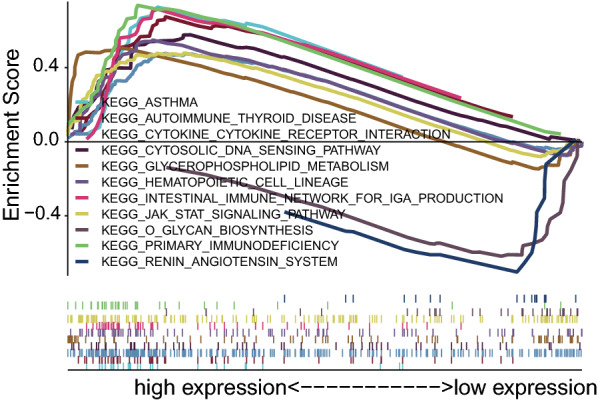
Enrichment plots from multiple GSEA

The differentially regulated pathways between high and low expression of HSPA7 groups were identified by GSEA and then the activated signaling pathways in KIRC were founded. The results with significant differences in enrichment (FDR < 0.25, NOM p < 0.05) in the MSigDB gene set (c2.cp.kegg.v6.2.symbols.gmt) were selected based on the NES and listed in Table 5. Figure 4 showed that renin angiotensin system, primary immunodeficiency, O-glycan biosynthesis, JAK-STAT signaling pathway, hematopoietic cell lineage, intestinal immune network for IgA production, glycerophospholipid metabolism, cytokine-cytokine receptor interaction, cytosolic DNA sensing pathway, autoimmune thyroid disease and asthma. GSEA analysis displayed the high expression of HSPA7 in KIRC were related to several tumor- and immune-related pathways.

**Table 5:** GSEA identifies an HSPA7-related signaling pathway

NAME	NES	NOM p-val	FDR q-val
KEGG_O_GLYCAN_BIOSYNTHESIS	2.17	0.00	0.02
KEGG_RENIN_ANGIOTENSIN_SYSTEM	2.21	0.00	0.02
KEGG_ALDOSTERONE_REGULATED_SODIUM_REABSORPTION	1.92	0.00	0.09
KEGG_PROXIMAL_TUBULE_BICARBONATE_RECLAMATION	1.93	0.00	0.09
KEGG_ARRHYTHMOGENIC_RIGHT_VENTRICULAR_CARDIOMYOPATHY_ARVC	1.87	0.01	0.10
KEGG_TIGHT_JUNCTION	1.89	0.01	0.10
KEGG_STEROID_BIOSYNTHESIS	1.94	0.00	0.11
KEGG_GLYCOLYSIS_GLUCONEOGENESIS	1.85	0.02	0.11
KEGG_CITRATE_CYCLE_TCA_CYCLE	1.95	0.00	0.12
KEGG_BIOSYNTHESIS_OF_UNSATURATED_FATTY_ACIDS	1.81	0.01	0.12
KEGG_VALINE_LEUCINE_AND_ISOLEUCINE_DEGRADATION	1.79	0.04	0.12
KEGG_N_GLYCAN_BIOSYNTHESIS	1.73	0.04	0.13
KEGG_PYRUVATE_METABOLISM	1.77	0.04	0.13
KEGG_THYROID_CANCER	1.71	0.03	0.13
KEGG_PROPANOATE_METABOLISM	1.79	0.04	0.13
KEGG_VIBRIO_CHOLERAE_INFECTION	1.73	0.05	0.13
KEGG_ADHERENS_JUNCTION	1.97	0.02	0.13
KEGG_SPHINGOLIPID_METABOLISM	1.73	0.02	0.14
KEGG_PENTOSE_PHOSPHATE_PATHWAY	1.74	0.01	0.15
KEGG_VASOPRESSIN_REGULATED_WATER_REABSORPTION	1.65	0.04	0.16
KEGG_MELANOMA	1.61	0.03	0.18
KEGG_LONG_TERM_DEPRESSION	1.56	0.03	0.19
KEGG_GLYCEROPHOSPHOLIPID_METABOLISM	2.26	0.00	0.01
KEGG_INTESTINAL_IMMUNE_NETWORK_FOR_IGA_PRODUCTION	2.15	0.01	0.03
KEGG_CYTOSOLIC_DNA_SENSING_PATHWAY	2.12	0.00	0.03
KEGG_ASTHMA	2.05	0.01	0.03
KEGG_AUTOIMMUNE_THYROID_DISEASE	2.05	0.01	0.03
KEGG_JAK_STAT_SIGNALING_PATHWAY	2.01	0.00	0.04
KEGG_PRIMARY_IMMUNODEFICIENCY	2.00	0.01	0.04
KEGG_CYTOKINE_CYTOKINE_RECEPTOR_INTERACTION	2.00	0.00	0.03
KEGG_HEMATOPOIETIC_CELL_LINEAGE	1.95	0.01	0.04
KEGG_NATURAL_KILLER_CELL_MEDIATED_CYTOTOXICITY	1.92	0.01	0.05
KEGG_HOMOLOGOUS_RECOMBINATION	1.90	0.01	0.05
KEGG_RIG_I_LIKE_RECEPTOR_SIGNALING_PATHWAY	1.87	0.02	0.06
KEGG_FC_EPSILON_RI_SIGNALING_PATHWAY	1.82	0.02	0.07
KEGG_NOD_LIKE_RECEPTOR_SIGNALING_PATHWAY	1.81	0.03	0.07
KEGG_ALLOGRAFT_REJECTION	1.80	0.04	0.07
KEGG_SNARE_INTERACTIONS_IN_VESICULAR_TRANSPORT	1.77	0.02	0.08
KEGG_ALPHA_LINOLENIC_ACID_METABOLISM	1.76	0.00	0.08
KEGG_GNRH_SIGNALING_PATHWAY	1.75	0.01	0.08
KEGG_ETHER_LIPID_METABOLISM	1.70	0.01	0.09
KEGG_PHOSPHATIDYLINOSITOL_SIGNALING_SYSTEM	1.67	0.04	0.09
KEGG_ABC_TRANSPORTERS	1.61	0.03	0.11
KEGG_LINOLEIC_ACID_METABOLISM	1.59	0.03	0.12
KEGG_HEDGEHOG_SIGNALING_PATHWAY	1.53	0.05	0.14
KEGG_ARACHIDONIC_ACID_METABOLISM	1.50	0.04	0.14
KEGG_VEGF_SIGNALING_PATHWAY	1.49	0.04	0.14

### HSPA7 expression correlated with immune cell infiltration in KIRC

**Fig. 5:**
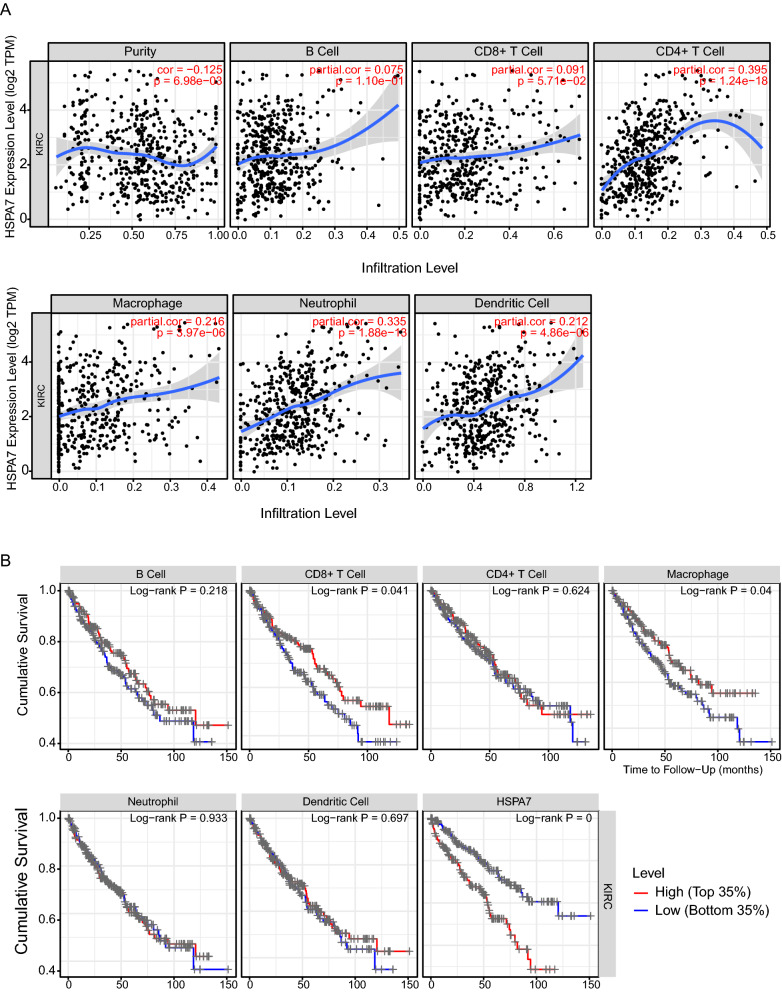
HSPA7 expression is correlated with the level of immune infiltration in KIRC. **A** HSPA7 expression is correlated with the level of immune infiltration in KIRC. **B** Kaplan–Meier plots of immune infiltration and HSPA7 expression levels in KIRC

Previous studies showed that lymph node metastasis and survival are independently predicted by the frequency of lymphocytes infiltrating in cancer patients. Also GSEA analysis displayed the high expression of HSPA7 in KIRC were related to immune-related pathways. Using TIMER database we investigated whether HSPA7 expression was correlated with six main infiltrating immune cells in KIRC. The result implied that expression of HSPA7 associated with CD4^+^ T cells (r = 0.395, p-value = 1.24e−18), Macrophage (r = 0.216, p-value = 3.97e−06), neutrophils (r = 0.335, p-value = 1.88e − 13) and Dendritic Cell (DC) (r = 0.212, p-value = 4.86e−06) (Fig. 5). The HSPA7 expression levels was also correlated with tumor purity (cor = 0.125, p-value = 6.98e−03). These results suggested that immune infiltration may serve as a important role in KIRC patient outcomes, and HSPA7 could modulate immune infiltrating cells into KIRC tissues.

## Discussion

Our study first reported that pseudogene HSPA7 was expressed highly in KIRC patients and can predict a poor prognosis. We showed that the up-regulated HSPA7 had statistical correlation with histological grade, clinical stage, M classification, T classification and overall survival in KIRC.

HSPA7 belongs to the heat shock protein 70 (HSP70) family, has long been considered as being a pseudogene which is transcribed in response to stress, but now suggest as a high homology to HSPA6 [22]. The HSP70 family is composed of about 13 members, including HSPA1L, HSPA2, HSPA5, HSPA6, HSPA7, HSPA8, HSPA12A, HSPA12B HSPA9, HSPA13 and HSPA14 [23, 24]. Accumulating data indicated that HSP70 family can play a causal role in cancer initiation. Evidence showed that HSPA1L can enhance cancer stem cell-like properties via regulating β-Catenin transcription and activating IGF1Rβ [25]. RNF144A interacted with HSPA2 can promote tumor growth and progression [26]. Down regulation of HSPA5 can promote ANXA1 and repress PSAT1 expression, which inhibiting the osteosarcoma cell proliferation and inducing cell apoptosis [27]. The expression of HSPA6 were found associated with the lung cancer [14], leukemia [16] and baldder cancer’s [28] migration, invasion and proliferation. HSPA8 could regulate the cell viability in pancreatic cancer cells [29] and serve as a molecular target in human hepatocellular carcinoma [30]. Overexpression of HSPA12A can suppresses renal carcinoma cell migration while promotes hepatocellular carcinoma growth [31]. Overexpression of HSPA12B can induce cisplatin resistance in non-small-cell lung cancer (NSCLC) [32]. HSPA9 is associating with survival and proliferation of thyroid carcinoma cells [33, 34]. Less information is available for HSPA7, HSPA13 and HSPA14 representing more distally related members of the HSP70 family. In our research we explored that highly-expressed HSPA7 is related to clinicopathological features of KIRC. Most importantly, univariate and multivariate Cox analyse demonstrated that HSPA7 expression is an independent prognostic indicator of KIRC survival and may be a promising biomarker for clinical applications. Through GSEA analysis, we found that the high expression of HSPA7 in KIRC may related to several immune pathways. HSPA7 expression was found to correlate with the degree of immune infiltration in KIRC through the TIMER database. Knowledge of the immune components has increased over the past decade. Several studies have reported that immune cells from infiltrating tumors are capable of acting as tumor suppressors or promoters in the tumor microenvironment. CD8+ T cells were reported to correlate with the improved survival of cancer patients [35, 36], while regulatory T cells and tumor-associated macrophages were correlated with the promotion of tumor development [37, 38]. Few studies have shown that the HSP70 family members can serve as immunes signature for prognosis of cancers [11]. And the role of Hsp70 in cell immune modulation has remained contentious, only several studies have shown that the HSP70 family members may related to the cell immune. For example, HSPA2 is related to the responses of bone marrow derived dendritic cells to LPS [39], HSPA8 is central at different key steps in the presentation of peptide antigens to CD4+ T cells, with a potential to regulate T and B cell activation and the final secretion of antibodies by plasma cells [40]. HSPA13 is critical for plasma cells development and may be a new target for eliminating pathologic plasma cells [41]. Our research showed that the expression of HSPA7 was significant correlated with macrophage, CD4^+^ T cells, neutrophils and dendritic cell infiltrating. With the subsequent Kaplan–Meier analysis we found that CD4^+^ T cells and macrophage cells can predict the KIRC patients prognosis.

## Conclusions

In summary, we explored that the pseudogenes HSPA7 is highly expressed in KIRC tumors and is correlated with tumor survival and progression. We implied that the expression level of HSPA7 was moderately positively associated with degree of macrophage, neutrophil, CD4^+^ T cells and DC infiltration, and weakly positively correlated with the degree of B cells and CD8^+^ T cells infiltration in KIRC tumor tissues. The pseudogene are believed as therapeutic targets or potential prognostic markers for KIRC tumor patients, while the detailed mechanism of pseudogene affect the KIRC patients prognosis is still to be explored.

## Supplementary Information

**Additional file 1: Table S1.** Clinical characteristics of KIRC patients (*n* = 20).

## Data Availability

The data generated or analyzed during this study are included in this article, or if absent are available from the corresponding author upon reasonable request.

## Abbreviations

KIRC: Kidney Renal Clear Cell Carcinoma
HSPA7: Heat Shock Protein Family A (Hsp70) Member 7
TIMER: Tumor Immunoassay Resource
RCC: Renal cell carcinoma
TCGA: The Cancer Genome Atlas
GSEA: Gene Set Enrichment Analysis
NES: Normalized enrichment score
OS: Overall survival
DC: Dendritic cell
