# Supplementary material for: Pseudogene HSPA7 is a poor prognostic biomarker in Kidney Renal Clear Cell Carcinoma (KIRC) and correlated with immune infiltrates
Source: Cancer Cell Int. 2021 Aug 19;21:435. doi: 10.1186/s12935-021-02141-1 (PMC8375184; doi:10.1186/s12935-021-02141-1)
Supplement: Supplementary file 1 — Additional file 1: Table S1. Clinical characteristics of KIRC patients (n = 20). [file 12935_2021_2141_MOESM1_ESM.docx]

| **Table S1 \| Clinical characteristics of KIRC patients (*n* = 20).** | | |
| --- | --- | --- |
| **Clinical characteristics** | **Total (20)** | **%** |
| **Age**  *<*60 | 1 | 5 |
| ≥60 | 19 | 95 |
| **Gender** |  |  |
| Female | 9 | 45 |
| Male | 11 | 55 |
| **Histologic grade** |  |  |
| G1–2 | 4 | 20 |
| G3–4 | 12 | 60 |
| Gx | 3 | 15 |
| NA | 1 | 5 |
| **Stage** |  |  |
| I–II | 3 | 15 |
| III–IV | 17 | 85 |
| **T classification** |  |  |
| T1–2 | 9 | 45 |
| T3–4 | 11 | 55 |
| **N classification** |  |  |
| N0 | 2 | 10 |
| N1 | 17 | 85 |
| Nx | 1 | 5 |
| **M classification** |  |  |
| M0 | 6 | 30 |
| M1 | 12 | 60 |
| Mx | 2 | 10 |
| **Vital status** |  |  |
| Deceased | 17 | 85 |
| Living | 3 | 15 |
